# Curcumin and Its Derivatives as Theranostic Agents in Alzheimer’s Disease: The Implication of Nanotechnology

**DOI:** 10.3390/ijms22010196

**Published:** 2020-12-27

**Authors:** Umair Shabbir, Momna Rubab, Akanksha Tyagi, Deog-Hwan Oh

**Affiliations:** Department of Food Science and Biotechnology, College of Agriculture and Life Sciences, Kangwon National University, Chuncheon 200-701, Korea; umair336@gmail.com (U.S.); rubab.momna@gmail.com (M.R.); akanksha.tyagi001@gmail.com (A.T.)

**Keywords:** neurodegenerative disorder, curcumin, nanoparticles, diagnosis, therapeutic

## Abstract

Curcumin is a polyphenolic natural compound with diverse and attractive biological properties, which may prevent or ameliorate pathological processes underlying age-related cognitive decline, Alzheimer’s disease (AD), dementia, or mode disorders. AD is a chronic neurodegenerative disorder that is known as one of the rapidly growing diseases, especially in the elderly population. Moreover, being the eminent cause of dementia, posing problems for families, societies as well a severe burden on the economy. There are no effective drugs to cure AD. Although curcumin and its derivatives have shown properties that can be considered useful in inhibiting the hallmarks of AD, however, they have low bioavailability. Furthermore, to combat diagnostic and therapeutic limitations, various nanoformulations have also been recognized as theranostic agents that can also enhance the pharmacokinetic properties of curcumin and other bioactive compounds. Nanocarriers have shown beneficial properties to deliver curcumin and other nutritional compounds against the blood-brain barrier to efficiently distribute them in the brain. This review spotlights the role and effectiveness of curcumin and its derivatives in AD. Besides, the gut metabolism of curcumin and the effects of nanoparticles and their possible activity as diagnostic and therapeutic agents in AD also discussed.

## 1. Introduction

Globally, mental disorders are one of the main reasons for disability and pointing concerns for public health issues [1]. In 2010, 16.2% of the world population consisted of people aged 65 or over, a figure that is expected to rise up to 26.9% by 2050. Increasing life expectancy highlights the importance of physical and mental health in old age. Aging is a very complex process that alters an individual’s normal functioning resulting in deterioration of biological functions, especially brain and cognitive functions over a long period. Previous studies have generated inconsistent findings of the prevalence of mental illness among older adults [2]. Due to their higher prevalence, mental problems are contributing to significant health, economic and social burden [3]. Previous studies tended to focus on selective disorders such as dementia or depression, implying that the entire range of mental disorders has been insufficiently addressed [4]. Dementia is a syndrome (or group of symptoms) that causes deterioration in behavior, ability to perform everyday activities, thinking, memory, and learning capacity, language, and judgement. Dementia is not a normal part of aging but generally affects old age people [5]. Among the different kinds of dementia, the most common form is Alzheimer’s disease (AD) [6] and may contribute to 60–80% of cases [7]. According to WHO [5], AD and other types of dementia are the 5th prominent reason for deaths in the world and become a growing public health concern, with about 10 million new cases every year across the world and it is estimated that the frequency will be doubled by 2030 and tripled by 2050 [8]. The conditions worsen over the years, to the point, people suffering from AD may have difficulty in remembering events, names, or recent conversations and can have depression in very early stages. Later these symptoms lead to behavioral changes, confusion, impaired communication, poor judgment, disorientation, and ultimately patients are facing difficulty in walking, swallowing, and speaking [7]. There are two major hallmarks of AD as follows; (1) development of amyloidal plaques outside the cells nerve due to cleavage of membrane-embedded proteins (amyloid precursor proteins: APP) into the neurotoxic single amyloidal units (amyloid-beta: Aβ) peptides during proteolytic processing by secretases enzymes, such as β and γ-secretase, and (2) formation of neurofibrillary tangles (NFTs) inside the nerve cells due to accumulation of paired helical filaments of hyperphosphorylated tau proteins [6]. Aβ oligomers are the small and soluble aggregates of Aβ-peptide are considered as the key pathogenic structures in AD [9,10,11]. Despite Aβ-tau hypothesis; vascular abnormalities, oxidative stress, neuroinflammation, mitochondrial damage, etc. are also contributing to the pathogenesis of AD [12].

Mitochondria is the essential component of a cell that plays role in cell energy production and pursuing the nutrient-sensing and growth signals within nerve cells [13]. Recently, studies have been exposed that oxidative stress in mitochondrial microglia has also played a part in the development of AD. Loss of mitochondrial membrane potential and elevated generation of reactive oxygen species (ROS) through various mechanisms have been observed in AD. Higher ROS in microglia cause inflammation and activating cell pathways of cell death [14]. Available medicinal treatments or strategies bring only symptomatic benefits, and still, there is no perfect/ideal cure for AD [15]. Therefore, the lack of effective pharmacology therapy has led researchers to seek alternative approaches to treat or prevent AD such as diet, because nutrients are important for everyday systematic function. Many epidemiological evidence suggest a strong correlation among lifestyle factors, diet, and the onset and consolidation of AD and other kinds of dementia. Moreover, it has been proven that metabolic syndromes and disorders like insulin resistance, obesity, cardiovascular diseases, diabetes, and AD are strongly related. Nutritional interventions and other preventive strategies can be effective approaches to stop or delay the risk of AD, cognitive decline and other non-psychiatric comorbidities. Many nutrients play a role in biochemical reactions; however, intake of a diet rich in probiotics, antioxidants, plant-based foods, ω-3 polyunsaturated fatty acids, soybeans, and nuts can be beneficial in mitigating AD. Furthermore, less consumption of animal-derived proteins, refined sugars, and low intake of saturated fats can also be useful in this regard [16]. Plant-based polyphenols (including curcumin) have been recognized as potent agents that can help lower down the effects of AD. Curcumin and its derivatives have potential to ameliorate the hallmarks of AD and other neurodegenerative diseases but still, clinical studies are required to prove this fact. This review summarizes the role of curcumin and its derivatives those have been beneficially associated with the upkeep of neurocognitive capacity and the risk of AD. Association of gut metabolism and curcumin to ameliorate the effects of AD and the effects of nanoformulations as theranostic agents (diagnosis and therapeutic) with the possible role and action in AD are also explored.

## 2. Curcumin

Curcumin (*Curcuma longa* L.) commonly known as turmeric belongs to Zingiberaceae (or ginger family) is a bright yellow plant pigment, a popular spice, and food additive widely used in South Asian and Middle Eastern countries [17]. Zingiberaceae is a family of flowering plants which is a rhizomatous herbaceous perennial known as Angiosperms. The powdered rhizome (of turmeric) is used in traditional medicine to cure various kinds of maladies as well as a coloring agent in beverage industries [18]. Compositionally, three main compounds of curcumin (1,7-bis[4-hydroxy-3-methoxyphenyl]-1,6-heptadiene-3,5-dione) are curcuminoid complex (80%), dimethoxy-curcumin (17%, 1,7-bis(3,4-dimethoxyphenyl)-1,6-heptadiene-3,5-dione) and bisdemethoxy-curcumin (3%, 1,7-Bis(4-hydroxyphenyl)-1,6-heptadiene-3,5-dione) [19]. Modern medicine has shown that curcumin exhibits a broad range of biological and pharmacological activities, including antioxidant [20], anti-inflammatory [21], anti-tumor and chemosensitizing [22], hepatoprotective [23], lipid-modifying [24] and neuroprotective [25] effects and are suggested to improve mental illnesses due to its ability to modulate numerous signaling molecules [8,26,27]. Many studies show that it is safe to consume 8 g per day of curcumin [28,29]. Therefore, many curcumin based products are available such as tablets, capsules, and as an additive to various energy drinks [27].

### 2.1. Curcumin and Aβ 

**Figure 1:**
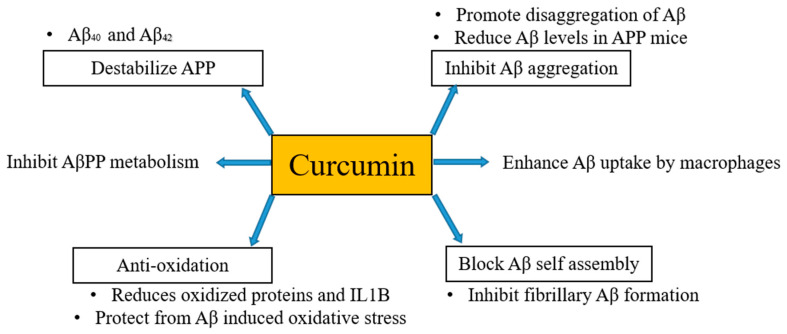
Anti-amyloid properties of curcumin. Curcumin regulates Aβ metabolism and inhibits Aβ aggregation in several ways.

It has been mentioned above and also evident from several studies that the formation of Aβ plaques is the beginning of the onset of AD. Thus, inhibition or hindrance of these plaques can be useful to deal with AD. Curcumin is proposed to be resistant to Aβ aggregation as has a higher binding affinity to Aβ [30] and its derivatives are also considered as principal candidates to prevent the aggregation of amyloid plaques. Qin et al. [31] in their work regarding detection strategies of AD disclosed that curcumin has potential to bind Aβ and iron in plaques through intermolecular hydrogen bonds without the combination of any other chemical linkages or bonding. Molecular chains of curcumin have symmetrical methoxyl and phenyl groups. These groups can easily bind amyloid plaques via hydrophobic interactions with nonpolar sections of the amyloid plaques. Further, these can be stabilized by di-ketone and hydroxyl groups of curcumin with polar sections of Aβ plaques by hydrogen bonding. Consequently, curcumin can be considered as potent candidate for binding and locating Aβ plaques in a brain suffering from AD [32]. Besides, in vivo studies have documented that curcumin inhibits the aggregation of Aβ and also reduces the size of deposits [33,34]. Curcumin destabilizes Aβ40 and Aβ42 [35] as well as pyrazoles and isoxazoles (derived from curcumin) upon binding with Aβ inhibits the metabolism of AβPP [36]. Curcumin also has the potential to block Aβ self-assembly by inhibiting Aβ formation [33]. Kim et al. [37] stated that curcumin protects human umbilical endothelial cells and PC12 cells (PC12 cell line is used in neuroscience research, such as studies on neuroprotection, neurotoxicity, and neuroinflammation [38]) from oxidative stress-induced by Aβ that helps to suppress the levels of oxidized proteins and interleukin-1β in the brains of APP mice. Fiala et al. [39] proposed that bone marrow-derived dendritic cells may correct immune defects and offer immunotherapy to the person suffering from AD by enhancing the Aβ uptake by macrophages. Curcumin regulates Aβ metabolism and inhibits Aβ aggregation in several ways, as shown in Figure 1.

Yang et al. [33] reported that curcumin reduced the aggregation of Aβ oligomers in vitro. Tetrahydrocurcumin, a metabolite of curcumin, increased the levels of ROS, decrease mitochondrial membrane potential, and protects human neurons from oligomeric Aβ induced toxicity [40]. On the other hand, a study conducted by Arjun et al. [41] claimed that curcumin did not hinder Aβ fibril formation, but relatively enhance the population of “off-pathways” pre-fibrillar aggregates and soluble oligomers that were non-toxic. They also stated that curcumin reduced the toxicities instigated by Aβ conformers such as fibrillar, pre-fibrillar, monomeric, and oligomeric Aβ and exhibited a non-specific neuroprotective impact. Moreover, the neuroprotective effect of curcumin might be membrane-mediated as it suppresses the cell membrane permeability instigated by Aβ aggregates. So, their findings suggest two pathways for curcumin’s neuroprotective ability (1) modification of Aβ aggregates towards the development of non-toxic aggregates, (2) amelioration of Aβ-instigated toxicity via non-specific pathways.

### 2.2. Curcumin and Glial Cells

**Figure 2:**
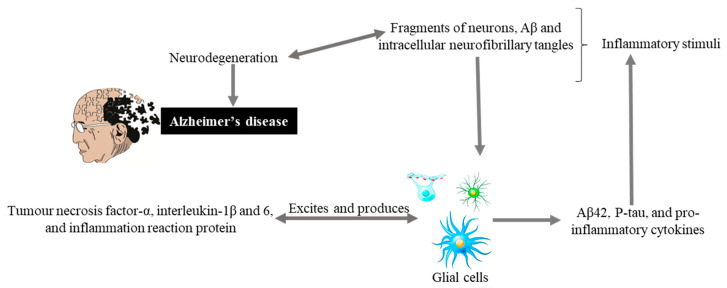
Hypothetical inflammation in AD. Inflammatory stimuli activate glial cells that produce tumor necrosis factor-α, interleukin-1β and 6, and inflammation reaction proteins. These can also excite glial cells that produce Aβ42, P-tau, and pro-inflammatory cytokines, and cycle is maintained that leads to neurodegeneration and Alzheimer’s disease.

Glial cells are non-neuronal cells (consisting of astrocytes, oligodendrocyte lineage cells as their significant components and especially microglia) from the immune system of the central nervous system (CNS) play major roles in modulating neural plasticity, maintaining homeostasis, and shaping brain development [42]. Microglia, being the primary immune cell of the CNS and due to highly responsive cell, reacts immediately against local injury, a multiplicity of brain pathologies, neuroinflammation, and immune surveillance [43]. Recently conducted studies on genetics have highlighted more about the importance of these cells as with the discovery of many polymorphisms in microglial-enriched genes. They are related to various neurological disorders such as amyotrophic lateral sclerosis, frontotemporal dementia, AD, schizophrenia, and autism [42]. Glial cells originate an inflammatory cascade under the influence of nuclear factor kappa-light-chain-enhancer of activated B cells (NF-κB) signaling. NF-κB seems to modulate the expression of many chemokines and cytokines, including interleukin, tumor, interferon, necrosis factor α, and inflammation reactive proteins. These proteins and cytokines through paracrine or autocrine pathways excite the glial cells for additional production of Aβ42, p-tau, and pro-inflammatory molecules that causing neurodegeneration (Figure 2).

Concerning this, many studies have reported that through inhibiting activation of the TLR4/MyD88/NF-κB signaling pathway and promoting M2 polarization, curcumin offers protection of neurons [44]. Liu et al. [45] documented that curcumin enhances the PPARγ activity and reduces the production of cytokines, astrocytes, and microglia through the inhibition of the NF-κB signaling that decreases the neuroinflammation in AD induced rats. Curcumin could regulate the production of CC motif ligand 2 and reduce c-Jun N-terminal kinase phosphorylation in astrocytoma cells [46]. Additionally, curcumin has the ability to enhance the RANTES (Regulated upon Activation, Normal T Cell Expressed and Presumably Secreted) expression in P1-3K signaling pathways, activated mitogen-activated protein kinase (MAPK), and in astrocytes so, ultimately shows neuroprotective activities in rats [47]. Tai et al. [48] exhibited that curcuminoid submicron particles can improve the pathological deficits and memory impairment in mouse with AD. Moreover, it decreases the astrogliosis and Aβ plaques in vivo and enhances the microglial Aβ phagocytosis in vitro. Thus, these are the main mechanisms that shows the beneficial properties of curcuminoid submicron particles in modulation of neuroinflammation. Curcumin at 160 ppm can reduce oxidative damage, significantly reduce Aβ and can inhibit markers of glial inflammation such as cytokine interleukin-1 and glial fibrillary acidic protein in AD transgenic mice [49]. Hence, curcumin has low toxicity and high efficacy, so can be a potential agent for AD treatment.

### 2.3. Curcumin and Tau Proteins

Tau proteins are associated with microtubules (major components of the cytoskeleton and abundantly present in neurons), found in the central and peripheral nervous system. Post-translational modifications (most notable phosphorylation) are deciding the localization of tau in neurons [50]. Same as other proteins associated with microtubule, tau also monitors intracellular trafficking and stabilizes microtubule integrity [51]. Conversely, in AD, tau losses their normal functioning to bind microtubules but start to bind each other’s which results in accumulated phosphorylation. Due to phosphorylated tau overall tubulin assembly starting to decrease that lead towards the development/formation of intraneuronal NFTs [52]. These NFTs remain intracellular and causing the death of neurons [53]. Moreover, impaired tau’s function increases the burden on the mitochondrial function that leads to ROS release and mitochondrial dysfunction [54]. Thus, therapeutic agents that can target tau pathology, not only repair abnormality in tau but also restrain a series of reactions caused by the abnormality of tau could play a vital role in the treatment of AD [55]. Studies have been confirmed that curcumin has these abilities that could decrease hyperphosphorylated tau aggregation and restrain tau hyperphosphorylation [56] by avoiding intracellular fibrillary tangles [57,58]. Curcumin can suppress tau protein dimmer formation and hyperphosphorylated tau protein oligomerization and prevents glycogen synthase kinase-3β activity in tau protein-induced AD mice. However, docosahexaenoic acid and curcumin (oral administration) inhibited insulin receptor substrate 1 and c-Jun N-terminal kinase (family of protein kinases that have a significant role in stress signaling pathways associated with neuronal plasticity, cell death, gene expression, and regulation of cellular senescence [59]) activities that suppressed hyperphophorylated tau protein levels [60]. Another mechanism of hyperphosphorylation inhibition by curcumin is reported by Huang et al. [61] which showed that human neuroblastoma SHSY5Y cells via the phosphate and tensin homologue/protein kinase B (Akt)/GSK-3 pathway which is induced by Aβ inhibits the tau pathologies.

### 2.4. Curcumin, Oxidative Stress and Metal Chelation

It has been reported that oxidative stress plays a vital role in the pathogenesis of neurological and age-related diseases. Activation of nuclear factor erythroid 2-related factor 2 (Nrf2) occurs during oxidative stress that modulates the expression of various antioxidant dense enzymes and exhibits protective effects for free radical-mediated neuronal damage [62]. Further, Nrf2 also regulate genes related to nerve growth factor signaling and autophagy [63]. Several lines of evidence have demonstrated activation of the Nrf2 pathway as a promising novel strategy for the management and prevention of diseases related to the brain. Curcumin through its antioxidant effect raises the levels of glutathione that suppresses formation of 3-nitrotyrosine (indicator of nitric oxide production, inflammation, and neural damage) [64]. Gao et al. [65] demonstrated curcumin loaded T807/triphenylphosphine-red blood cell nanoparticles in AD model mice and found relieved symptoms of AD by mitigating mitochondrial oxidative stress and neural death in vitro and in vivo. Curcumin exhibits metal chelation properties that are due to its two methoxyphenol groups linked with a β di-ketone linker, which helps in scavenging hydroxyl radicals preserve glutathione and superoxide that ultimately suppresses oxidative damage. Additionally, it is also a selective activator of Nrf2/Keap1/ARE, which trigger heme oxygenase-1 (a redox-sensitive inducible protein) and relieves oxidative stress-mediated neuronal injury [66]. As well, curcumin shields astrocytes (as activation of astrocytes is one of the key pathological hallmarks of neural disorders) from oxidative stress and mitochondria impairment. It has a defensive action on astrocytes by its negative consequence on apoptosis and reactive astrogliosis [67]. It has been documented that curcumin prevents astrogliosis in astrocytes, mitochondrial dysfunction, and caspase 1-dependent inflammation that ultimately hampers the mitochondria-dependent/independent apoptosis caused by oxidative damage [68]. Maiti et al. [69] observed that relative to natural curcumin, solid lipid curcumin particles were more permeable, useful on Aβ plaques and produced a more considerable decrease of pyknotic or tangle-like, neurons in the prefrontal cortex, CA1, and CA3 areas of the hippocampus.

## 3. Limitations with Chemical Properties

Administration of curcumin can exhibit neuroprotective effects but due to blood-brain barrier (interaction with specific efflux transporters) or due to the presence of hydroxyl group, β-diketone moiety, and active methylene group in its structure, and its lipophilic nature show low pharmacokinetics/bioavailability [70,71]. The existence of hydroxyl groups in curcumin’s structure make it to metabolize by the kidney and liver enzymes rapidly; that’s why indicates poor absorption. Additionally, curcumin is very unstable in most body fluids like water, so, it is recommended to mix with milk or oil before consumption to increase absorption and metabolism. Thus, these properties making its use limited, leading to a challenge for the disease treatment in in vivo studies as a neuroprotective agent. Hence new formulations of curcumin are urgently needed, mainly to increase curcumin’s solubility for AD treatment. Recently, several researchers have been reported that, combining curcumin with other bioactive compounds could be useful against the treatment of AD. Lin et al. [72] combined curcumin and berberine treatment and found significant results (synergistically). They reported reduced soluble Aβ_(1–42)_ peptide production, oxidative stress and inflammatory responses in both the hippocampus and cortex of AD mice. Furthermore, Alamro et al. [73] studied the effect of curcumin and vitamin D3 on primary cortical neuronal cultures exposed to Aβ_1–42_ toxicity. Experimental treatments with Aβ peptide (1–42) showed an increase in lipid peroxidation products in the existence of curcumin and vitamin D_3_. Biochemical assays for oxidative stress including glutathione, lipid peroxidation, catalase, superoxide dismutase and glutathione S-transferase were raised significantly in the presence of vitamin D_3_ and curcumin. This study exhibited that curcumin and vitamin D_3_ treatment showed better recovery of neuronal cells. Moreover, the upregulation of neurotrophic growth factor levels was also observed.

## 4. Curcumin, Gut Microbiota and AD

**Figure 3:**
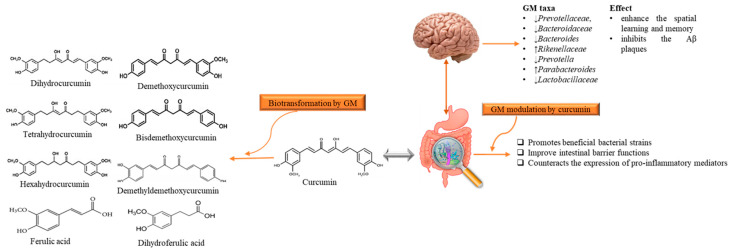
The reciprocal interaction between curcumin and Gut microbiota. Biotransformation of curcumin occurs due to gut microbiota that converts it into several metabolites through pathways like demethylation, reduction, acetylation, hydroxylation, and demethoxylation. These metabolites through gut-brain axis show antioxidant, anti-inflammatory, and neuroprotective effects. While, gut microbiota modulation alters the microbial abundance, diversity and composition, which also exert health benefits in Alzheimer disease-induced rats, indirectly. GM: gut microbiota.

Although AD is a neurodegenerative disease, however, scientists are considering a new hypothesis that despite the limited bioavailability of curcumin in gut, it has an indirect influence on the CNS that is due to microbiota-gut-brain axis [74]. It has been stated that gut bacterial metabolites employ their neuroprotective effects in several neurodegenerative disorders like AD [74,75,76]. Microbiota–gut-brain axis is a complex but bidirectional system in which gut microbiota (GM) and its composition represent a factor that preserves and determines the brain health [77,78]. Recently Sun et al. [79] revealed that the interaction between curcumin and GM is bidirectional as GM transform curcumin while curcumin influences on the abundance of GM. It has been found that administration of curcumin tends to enhance the spatial learning and memory abilities as well as inhibits the Aβ plaques in the hippocampus of APP/PS1 mice. Apart from this, curcumin significantly alters the composition of GM taxa such as *Prevotellaceae*, *Bacteroidaceae*, *Lactobacillaceae*, and *Rikenellaceae* at family level *Bacteroides, Prevotella*, and *Parabacteroides* at the genus level, several of them are considered to be associated with AD development [79]. While the GM metabolite curcumin through demethylation, reduction, acetylation, hydroxylation, and demethoxylation [80]. The produced metabolites have been documented to show neuroprotective effects in AD-induced mice [79] (Figure 3). It has been considered that gut metabolism can indirectly pose neuroprotective abilities via modulation of gut-brain axis (GBX) [75,81]. These findings not only enlighten the paradox between the low bioavailability of curcumin and its pharmacological effects, but also claim that GM might act as a useful source for microbiome-targeting therapies for AD. The low oral bioavailability of curcumin may be speculated as a plausible factor that limits its effects in humans [68]. Thus, utilization of several approaches can improve the bioavailability and its therapeutics as Annunziata et al. [82] proposed that fermentation is a natural strategy with minimum environmental impacts to increase the bioavailability of bioactive compounds (e.g., curcumin) that may help to cope up with AD. Fermentation mainly enhances the solubility and converts it to activated form that the body readily utilizes it for proper therapeutic effects. There are different types of fermentation (including lactic acid fermentation, alcohol fermentation, and acid fermentation), among them lactic acid fermentation considered as better because it is not contributing to cytotoxicity [83]. On the other hand, microencapsulation is a new strategy to improve the availability, delivery and therapeutic effects [84]. In microencapsulation, substances are incorporated in microscopic capsules that can measure from millimeter to micrometer, consist continuous films of coating material [85]. Microencapsulation enhances the solubility of curcumin in the aqueous solution and protect it during the gastrointestinal tract interact as well as increase the residence of curcumin and other substances in the intestine with the promotion of endocytosis [86]. Besides, Reddy et al. [75] suggested that nanoencapsulation is the encapsulation of substances at the nanoscale, improves the solubility, stability, target specificity, and drug release of the substance. It is considered advantageous as compared to microencapsulation as they have ultrathin layers that improve mass transport of substances to the islets and also reduces the volume of material [87]. Positive effects of these approaches in cell and animal models have shown positive results, but studies related to human are still lacking.

## 5. Curcumin Derivatives

To overcome the bioavailability issues, curcumin-based compounds have been synthesized. Designing and synthesis of novel compounds have shown a better pharmacokinetic profile, target selectivity, water solubility, and stability. Structural modification of molecules (e.g., curcumin) reduce the molecular size or increase the lipophilicity; the blood-brain barrier penetration can be enhanced by focusing on reducing the efflux transport, through improving the transcellular diffusion permeability [88]. Curcumin derivatives have shown the increased anti-neuroinflammation, anti-amyloidogenic, antioxidative, and tau formation inhibitory activities as compared to natural curcumin [89]. Modifications also amplify the mitochondrial-protective effects. Similarly, curcumin pyrazole derivatives (e.g., C1-C6 and CNB-001) have exhibited more defensive properties on mitochondrial dysfunction, and its related abnormalities by enhancing nuclear translocation of Nrf2, attenuating intracellular ROS, and inhibiting the ΔΨm (mitochondrial membrane potential) loss [90]. It has been documented that micelles of curcumin are beneficial in inhibiting oxidative stress and mitochondrial swelling than natural curcumin [91].

Further, the hybridization of different compounds with curcumin is another approach to combat its imperfection. It has been revealed that melatonin and curcumin hybridization can ameliorate the curcumin bioavailability, its functionality and can pass the blood-brain barrier. Thus, hybridization could be promising and significant approach in neuroprotective therapy [92,93]. In addition, bioconjugates of curcumin like di-glutamoyl esters, di-valinoyl, and di-demethylenated piperoyl enhance neuroprotective effects against mitochondrial dysfunction, damage and nitrosative stress [94]. Many studies have been conducted to modify curcumin’s structure to analyze their activity against AD and other neuro-related diseases. Curcumin derivatives, those have been confirmed to cross the blood-brain barrier and showed activity against AD hall markers are documented in Table 1. These studies have provided important details about the design and synthesis of curcumin and its derivatives.

## 6. Blood-Brain Barrier and Drug Delivery

**Figure 4:**
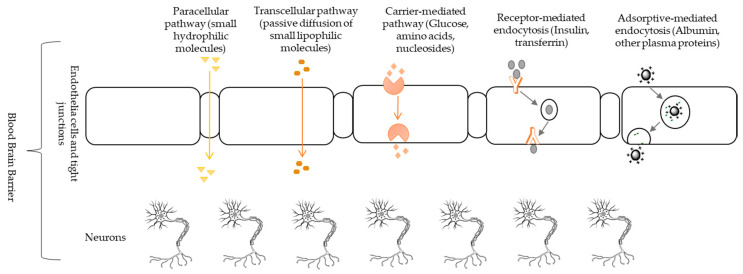
Main pathways for nanoparticles to cross the blood-brain barrier. Penetration of charged molecules (including cationic nanoparticles: liposomes and gold nanoparticles) depends on adsorptive-mediated endocytosis while molecules with high hydrophilicity (like transferrin conjugated nanoparticles, liposomes, poly(lactic-co-glycolic acid/poly (lactic acid)) needs receptor-mediated endocytosis. Carrier-mediated pathway is a substrate-specific process while small hydrophilic molecules penetrate through paracellular pathway. Passive diffusion of small lipophilic molecules, e.g., gold nanoparticles occur through transcellular pathway.

The blood-brain barrier is a highly selective semipermeable border of endothelial cells that is a border against circulating foreign substances from non-selectively crossing into the extracellular fluid of the brain. It is a hurdle against the delivery of therapeutic or diagnostic agents to the neurons residing in the brain [111]. Multiple ways have been reported for bio-macromolecules and small molecules to penetrate through the blood-brain barrier [112]. These pathways are generally driven by the concentration gradient of the substrates with the assistance of suitable transporters [113]. There are different ways to pass through the blood-brain barrier for small molecules so they can spread into the brain parenchyma (functional tissue, made up of neurons and glial cells) under physiological conditions (Figure 4) but dependent on their characteristics [113]. (1) for small hydrophilic molecules, paracellular transportation through an aqueous pathway is specific. It can transfer substances across the epithelium by passing through the intercellular space between the cells [112]. (2) the transcellular diffusion of small lipophilic molecules, those reach to the brain tissues through a non-saturable mechanism [114]. A certain amount of lipophilic drugs with molecular weight less than 500 Da can penetrate through the blood-brain barrier. In contrast to diffusion through the hydrophilic membrane, diffusion through lipophilic membranes is strongly dependent on the correlating features of surface area, molecular weight, and molecular volume of a substance [115]. (3) the carrier-mediated pathway, endogenous transporter or substrate-specific process in which the remaining small molecules reach to the brain [116], substances pass through the basolateral membrane and apical membrane. In the carrier-mediated pathway, proteins (ATP-binding cassette, glucose carrier, and LT1) are used to transport hydrophilic molecules through facilitated diffusion or active transport [117]. (4) the receptor-mediated transportation uses the vesicular trafficking of brain endothelial cells to transport some types of protein like leptin, insulin, lipoproteins, and transferrin [118]. D’Souza [117] recommended receptor-mediated endocytosis for the delivery of the targeted drug. Binding of ligands to the receptors triggers the internalization in receptor-mediated endocytosis. Thus, nanoformulations composed of ligands can transport across the membrane [119]. (5) Endothelial cells have negatively charged plasma membranes, positively charged molecules bind with membranes and cross blood-brain barrier by adsorptive-mediated endocytosis [120]. Majority of substances penetrate the brain through the carrier-mediated pathway, receptor-mediated pathway, and adsorptive-mediated pathway as structures of molecule possesses high affinity for a particular receptor, charged membranes or carrier found in blood-brain barrier [121].

Molecular Trojan horses and nanomedicines are the emerging and novel strategies used for the drug-delivery through the blood-brain barrier [122]. The specific properties of nanomaterials like prolonged blood circulation, non-toxicity, biocompatibility, and reduced size have been exploited for the creation/development of evolving delivery platforms that can facilitate the passage of therapeutic/diagnostic drugs to the brain [123].

## 7. Nanoformulations and Their Role in AD

In recent years, nanotechnology-based drug delivery systems have gained substantial attention because of their promising potential to prolong the residence and circulation of drugs in the blood and also enhance the drug’s aqueous stability and solubility. In addition, enhances the ability of drugs to cross the physiological barriers, that results in the enhancement of bioavailability [124].

### 7.1. Nanoparticles/Nanosensors as Theranostic Agents

Detection of AD during the early stages or the phase of the illness will offer great relief to patients suffering from AD, their friend and families, along with the economy [125]. Thus, it is the need of time to create multi-mode devices capable of simultaneous monitoring of many biomarkers that can stimulate fast, low cost, and reliable diagnosis [126]. Neurotransmitters are suitable biomarkers for various neurological disorders, such as AD. Several analytical techniques, such as microdialysis, capillary electrophoresis and electrochemistry are generally used to diagnose neurotransmitters of AD. However, researchers are still striving to discover an easy and accurate method to detect neurotransmitters of AD before devastating symptoms begin. In the last decade, biosensors have gained popularity as an attractive alternative method, and valuable efforts have been successfully made by scientists for the development of viable biosensors to propose a new analytical platform [127]. Biosensors are devices used for the detection of a chemical substance. They integrate a biochemical binding component with a signal conversion part and recently, several researchers have investigated the use of unique materials for developing biosensor for detection of different biomarkers of AD [128,129]. With the advancement in nanotechnology, nano-bio sensors have many prospective benefits as compared to other analytical or clinical methods. This advancement leads to improved assay efficiency, decrease in diagnostic testing costs, resourcefulness, and ability to deliver family general practitioners with molecular diagnostic devices [130]. Furthermore, the incorporation with nanocarriers has also been documented to enhance the conductivity and catalytic properties of the transducer. It happens while facilitating the immobilization of a large number of biological recognition elements because of the large surface area of nanoparticles [131]. The most common nanoparticle formulations that have a significant impact in the diagnosis and therapy of AD include polymeric nanoparticles, protein-based nanoparticle, gold nanoparticle, polysaccharide-based nanoparticle, selenium nanoparticle, gadolinium nanoparticle, etc. [132]. Recently, carbon nanomaterials, including carbon nanostructures, carbon dots, carbon nanotubes, graphite, graphene, and fullerene have received considerable significance [133]. After crossing the blood-brain barrier, nanoparticles can bind monomers and oligomers of Aβ so, specific inhibition of neurotoxicants can help to ameliorate AD. Surface functionalization or chemical modification of these nanocarriers may improve the aqueous solubility, and therefore, facilitating their utilization to treat the neurodegenerative disorders. Moreover, the concentration of drugs or conjugation of drugs with nanocarriers can be considerably used as an effective neuro-drug delivery system [134]. Mostly used nanomaterials that can penetrate through the blood-brain barrier and have diagnostic/therapeutic values are depicted in Table 2.

### 7.2. Curcumin Loaded with Nanoformulations and Their Therapeutic Effects in AD

Nano-delivery systems such as polymeric nanoparticles, peptide carriers, micelles, liposomes, cyclodextrins, conjugates, lipidic nanoparticles, emulsions, and solid dispersions have been extensively studied for improving the overall bioavailability of curcumin for enhanced brain delivery [149]. The main characteristic and transport mechanism for brain uptake and blood-brain barrier crossing of the main curcumin conjugated nanocarriers are exhibited in Table 3. Curcumin loaded with poly(lactic-co-glycolic acid; PLGA)-poly(ethylene glycol) nanoparticles permeate through the impaired blood-brain barrier and diffused efficiently through the brain parenchyma. Additionally, were localized in brain regions with AD, and exhibited a protecting impact in the injured neonatal brain [150]. Recently, a study stated the neuroprotective effect of red blood cell membrane-coated PLGA particles bearing T807 molecules attached to the red blood cell membrane surface (T807/RPCNP) loaded with curcumin. A stabilized and sustained curcumin release was observed. Synergistic effects of T807, T807/RPCNP showed effective penetration across the blood-brain barrier, and they also exhibited a high binding affinity to hyperphosphorylated tau in nerve cells where they block multiple critical pathways in tau-associated AD pathogenesis. Additionally, curcumin loaded T807/RPCNP nanoparticles could relieve AD symptoms by reducing phosphorylated-tau levels and inhibiting the neuronal death both in vitro and in vivo [151]. Curcumin nanoformulation by altering the surface of the PLGA polymer and encapsulation of selenium nanoparticles could decrease the Aβ load and inflammations in the brains samples of AD mice and treated the memory loss of the model mice. Moreover, the histopathological images of animal tissues displayed no deceptive abnormalities [32]. Zhang et al. [152] developed hydroxypropyl-β-cyclodextrin-encapsulated curcumin complexes (CUR/HP-β-CD inclusion complexes), and curcumin encapsulated chitosan-coated PLGA nanoparticles (CUR-CS-PLGA-NPs) and compared their effects through intranasal administration. CUR/HP-β-CD inclusion complexes (in vitro) showed stability under physiological parameters and also showed a higher cellular uptake level of curcumin as compared to CUR-CS-PLGA-NPs. Further, both formulations behaved to reduce cellular cytotoxicity and exhibited antioxidant and anti-inflammatory effects. in vivo, after intranasal administration, the area under cover values of curcumin in the brain and plasma of the CUR/HP-β-CD inclusion complex group also showed higher values. So, CUR/HP-β-CD inclusion complexes presented better properties for application in AD than CUR-CS-PLGA-NPs.

Recently, Yavarpour et al. [153] evaluated the neuroprotective effects of curcumin loaded lipid-core nanocapsules in a model of AD induced aged female mice. The results of this study showed that curcumin loaded lipid-core nanocapsules displayed significant neuroprotection against Aβ1-42-induced behavioral and neurochemical changes in a model of AD. In another study, Sadegh et al. [154] documented the nanostructured lipid carriers with curcumin targeted the oxidative stress parameters (ADP/ATP ratio, lipid peroxidation, and ROS formation) in the hippocampal tissue resulting in improved memory conditions. Moreover, histopathological studies showed the potential of nanostructured lipid carriers with curcumin in suppressing the hallmarks of Aβ in AD in vitro (animal model). Sadegh et al. [155] reported another study by targeting the neuroprotective effect of nanostructured lipid carriers and solid lipid nanoparticles loaded with curcumin. This study showed that both nanoparticle techniques have higher bioavailability in the brain. They showed that 2,2-diphenyl-1-picrylhydrazyl free radical scavenging preparation processes did not exhibit any significant effect on the antioxidant activity of curcumin. Furthermore, curcumin loaded with chitosan and bovine serum albumin nanoparticles effectively enhanced the drug permeation across the blood-brain barrier, accelerated the phagocytosis of the Aβ peptide and further endorsed the activation of microglia. Additionally, repressed the TLR4-MAPK/NF-κB signaling pathway and downregulated M1 macrophage polarization [156]. The nanomaterials discussed in this study have been demonstrated to ameliorate the availability of curcumin and can potently enhance neuronal differentiation and reverse Aβ-induced learning and memory deficits.

### 7.3. Curcumin Derivatives, Curcumin and Its Nanoformulations Based Diagnostic Properties

Curcumin has properties like natural fluorescence, lipophilicity, and high binding affinity to Aβ, making it able for early diagnostic probe/plaque labelling fluorochrome [166]. It has been documented that it fluoresces yellow/green under a violet/blue (436 nm) light. These natural fluorescent qualities of curcumin have been explored in many studies for diagnostic purpose, which make it to absorb light at about 420 nm and discharges fluorescence at around 530 nm in aqueous solutions [167]. During the last two decades, comprehensive research studies have been conducted to develop curcumin-based probes for targeting Aβ with positron emission tomography, with existing imaging modalities, magnetic resonance imaging, near-infrared fluorescence and two-photon microscopy [168]. Si et al. [169] worked on the near-infrared fluorescence imaging probes of Aβ plaques for the early diagnosis of AD. Curcumin derivative dyes showed a considerably significant improvement in its fluorescence intensity after binding to Aβ plaques. Furthermore, in vitro and in vivo fluorescence imaging of Aβ plaques strained with one of the dyes exhibited that the compound was a potential probe to detect Aβ plaques in AD. Chibhabha et al. [170] developed anionic and water-soluble DSPE-PEG2000 curcumin polymeric micelles (also referred as curcumin micelles) those can detect both brain and retinal Aβ plaques. Another study showed that the curcumin-Ni electrode reported high sensitivity and wide detection range from 0.001 to 10 nM Aβ oligomer. This electropolymerized curcumin pledge the detection of Aβ oligomer at lower concentration and therefore, can be a prospective tool for the early detection of AD [31]. Singh et al. [171] proposed curcumin encapsulated Pluronic F127 nanoparticles (FCur NPs) and compared their blood-brain barrier penetration of free curcumin and FCur NPs. The FCur NPs displayed 6.5-fold more potent fluoresce intensity in the brain of mice than free curcumin. Future, in vitro comparison with Congo red revealed that encapsulated curcumin maintains its ability to bind to Aβ plaques. FCur NPs also exhibited antioxidant and antiapoptotic activity when compared to free curcumin. The combination of in vitro and in vivo results suggests the potential utility of the inexpensive FCur NPs as a theranostic agent for AD. Hence, it can be proposed from the mentioned studies that curcumin and its nanoformulations based techniques cannot only be used for therapeutic purpose but also for diagnostic purpose.

## 8. Safety and Limitations of Nano-Based Strategies as Theranostic Agents

Several studies have proved the activity of nanoparticles against AD, but there are still many challenges that are concerning their application for biomedical purposes. These concerns are: (1) The short-term toxicity has been observed in many studies. (2) Most of the developed nanoparticles do not have long blood residence time that is why a large portion of them accumulate in the liver and spleen and unable to cross the blood-brain barrier. (3) To reduce the unintended adverse effects on target cells and organs, one acceptable biodegradable polymer that can increase the effectiveness of nanocarriers require. (4) Combination of nanotechnology and innovative high-tech methodologies (i.e., nano-chips or constructing implantable system/ multi-drug delivery processors) can help to strengthen drug delivery efficiency. (5) Nanoparticles those have a specific target for AD than other neurological diseases.

In diagnosis, stability and reproducibility of nanoparticles due to scalable and routine production of the bio-sensing system are also challenging. Apart from this, most studies have been conducted in artificial specimens or buffers, and real sample studies are minimal [172]. Since multiple hallmarks contributing to AD and the detection of these markers is very vital, that is still sorely missing. Thus, it is necessary to ensure a sensitive and accurate diagnosis system for the detection of those biomarkers. Some challenges are stated after. (1) need for the inclusion of one biosensor that can detect all of the pathological pathways. (2) new and improved diagnostic agents for easy, early and proper detection of AD. (3) Cost-effective techniques for early detection of hallmarks of AD.

## 9. Conclusions and Perspective

Curcumin is one of the most studied polyphenols in the spice turmeric, exhibiting complex and multifaceted activities. There has been much evidence on the protective effect of curcumin and its derivatives on the development of AD. Since AD is the most common form of dementia that occur mainly due to the formation/deposition of Aβ plaques and NFTs inside the nerve cells, the person suffering from AD can face many complications related to memory and brain health. Prevalence of AD posing a significant burden not only to patients but also their families due to the non-availability of an effective cure. A plethora of research work has been and is still being conducted to elucidate the complexities of AD pathology. The health-promoting effect of curcumin and its derivatives has been well documented in studies. Curcumin has shown therapeutic and diagnostic properties, but due to low bioavailability in the brain due to the blood-brain barrier, use is minimal. Therefore, curcumin-based compounds or nanoparticles have been synthesized. Designing and synthesis of curcumin-based compounds have shown a better pharmacokinetic profile and target selectivity. Additionally, nanostructured carriers have revealed the excellent encapsulating ability with improved physicochemical stability to penetrate against the blood-brain barrier. Nanocarriers as drug delivery carriers have exhibited many features such as controllable delivery, loading bioactive compounds with synergistic effects and also diagnostic properties. Although, nanoformulations conjugated with curcumin have shown many benefits against neurodegeneration but still need more to explore. In the future, nanoparticles with long blood residence time having less unintended adverse effects on target cells and organs with easy, early and proper detection are needed to be developed. Other than nanoformulations, microencapsulation also considered as a good strategy that can be used as a delivery vehicle to transport curcumin across the blood-brain barrier. Besides, due to GBX researcher are exhibiting new hypothesis that metabolites of curcumin can indirectly exhibit anti-inflammatory and antioxidative properties in AD. Taken together, current research suggests that curcumin is one of the most promising and exciting compounds for the development of AD therapeutics.

Although curcumin has multifaceted biological activity in the development of AD, however, more research needs to be done to improve the bioavailability of curcumin. Curcumin derivatives showed better bioavailability and theranostic properties as compared to natural curcumin and to increase the bioavailability of curcumin during gut metabolism, fermentation of curcumin before consumption can help improve the bioavailability. Microencapsulation and nanoencapsulation are also good options but nanoencapsulation showed better results in sense of solubility, stability and drug release than microencapsulation to enhance bioavailability in the context of a clinical study to treat AD. Moreover, the development of the early diagnosis of AD and new curcumin formulations are an active area of research. If curcumin is confirmed to show the same efficacy in humans as in vitro and in vivo studies, the disease-modifying treatment of AD is a worthwhile possibility.

## Figures and Tables

**Table 1:** Modified curcumin’s structure and their possible role against Alzheimer’s disease.

Modification of Curcumin	Neuroprotective Action	Study	References
Diketone replaced with pyrazole	Act as inhibitor against Aβ aggregation	In vitro and In vivo	[95]
Two aromatic rings connected by a nitrogen-containing bridge	Hinder extracellular amyloid toxicity	In vivo (rat hippocampal neurons)	[96]
Substituted derivatives of Dimethylaminomethyl (which have a large steric hindrance), to the ortho position of the hydroxy groups	Block the self-aggregation of Aβ	In vitro	[97]
Enol form of the compound Methoxycarbonylethyl group at the C-4 position	Significantly attenuation of the cell toxicity of Aβ High affinity for Aβ aggregation	In vitro and In vivo	[98]
Demethoxycurcumin	Effect on Aβ precursor protein through the internal ribosome entry sites	In vitro	[99]
At least one enone group in the spacer between aryl rings and an unsaturated carbon spacer between aryl rings. Methoxyl and hydroxyl substitutions in the meta and para-positions on the aryl rings	Act as inhibitor against Aβ aggregation	In vitro and In vivo	[100]
Monogalactose group	Inhibit Aβ peptide aggregation	In vitro	[101]
Contain more hydrophilic hydroxyl groups	Upregulate Neprilysin	In vitro and In vivo	[102]
Side aryl rings: 4-Hydroxy-3-methoxyphenyl as A ring and 4-Benzyloxyphenyl or para-tolyl as B ring	Inhibit β-secretase	In vitro docking	[103]
Half side of curcumin’s structure	Protect against Aβ toxicity through nematode ortholog of Nrf2	In vitro	[104]
Gd(III)(diethylenetriaminepentaacetate) and tert-butyl (2-propionamidoethyl)carbamate	Redirect metaltriggered Aβ aggregation	In vitro	[105]
4-Hydroxyl group	Protect from Aβ proteins (Aβ_1–42_)	In vitro	[106]
Hydroxyl group	Block the self-aggregation of Aβ	In vitro	[107]
4,6-Bis((E)-4-(1*H*-imidazol-1-yl)styryl)-2,2-difluoro-2*H*-1,3,2-dioxaborinin-1-ium-2-uide	Lowers Aβ levels in conditioned media and reduces oligomeric amyloid levels in the cells, Attenuates the maturation of APP in the secretory pathway, Inhibits β-secretase	In vitro and In vivo	[108]
Feruloyl-donepezil hybrid	Ability to modify the kinetics of Aβ fibril formation	In vitro In vivo	[109]
TML-6	Inhibit the synthesis of the APP and Aβ, upregulate Apo E, suppress NF-κB and mTOR, and increase the activity of the antioxidative Nrf2 gene	In vivo	[110]

Aβ: amyloid-beta, Nrf2: nuclear factor erythroid 2-related factor 2, APP: amyloid precursor protein.

**Table 2:** Different types of nanoformulations/biosensors in the diagnosis/therapy of AD.

Nanostructure	Diameter	Application	Role in AD	Study	References
Thioflavin-T	250–300 nm	Diagnostic	Identify Aβ in senile plaques	in vitro and In vivo rats and mice	[135,136]
		Therapeutic	Inhibit Aβ_42_ fibrillogenesis and disaggregate amyloid fibrils Exhibit cytoprotective effects against Aβ42 fibrillation-induced cytotoxicity	In vitro and In vivo	[137]
Gold	40–50 nm	Diagnostic	Targeted finding of tau protein in cerebrospinal fluid recognise the Aβ complexes	In vitro	[138,139]
		Therapeutic	Inhibition of Aβ aggregation, dissociate Aβ fibrils Decrease Aβ-mediated peroxidase activity and Aβ-induced cytotoxicity, acquisition and retention of spatial learning and memory	In vivo rats	[140,141]
Liposome	100–210 nm	Diagnostic	Target of amyloid plaques	In vitro	[142]
		Therapeutic	Inhibit the apoptosis of Aβ_1–42_, decrease lipid peroxidation level, acetylcholinesterase activity Prevents Aβ plaque formation	In vitro and In vivo rats	[143]
Polymeric based (natural and synthetic)	40–500 nm	Diagnostic	Identify Aβ and tau protein	In vitro and In vivo	[132]
		Therapeutic	Iinternalize successfully by oligodendrocytes, astrocytes, microglial cells, and neurons Destroy senile plaques and improve the memory impairment	In vitro and In vivo	[126,132]
Carbon based	1 nm to 10 s of nm	Diagnostic	Asses phosphorylated tau protein, tau protein, beta-amyloid40, and beta-amyloid42	In vitro and In vivo rats	[126]
		Therapeutic	Potential of decreasing Aβ	In vitro and In vivo rats	[126]
Gadolinium	2–15 nm	Diagnostic	Specifically, target amyloid fibrils for early detection of amyloid deposits	In vitro and In vivo mouse and mice	[144]
Quantum dots		Diagnostic	Identify the APP and Aβ plaque with enhancing sensibility compared with traditional fluoro immune assay	In vivo mice	[145]
Selenium	<200 nm	Therapeutic	Decrease Aβ plaques aggregation and decrease inflammations, Displays no deceptive abnormalities in histopathological images In vivo	In vitro and In vivo	[32]
Iron chelators	10–20 nm	Therapeutic	Detection of AD in early stages. Elevated brain iron is associated with cognitive decline. Conservative iron chelation with a high blood-brain barrier penetrant drug may slow the rate of neurodegeneration	In vitro and In vivo	[146,147]
Cerium	<50 nm	Therapeutic	Reduce the aggregation of Aβ, inhibit the formation of α-syn amyloid fibrils- associated cytotoxicity and reduce the oxidative stress	In vitro and In vivo	[148]

Aβ: amyloid-beta, AD: Alzheimer’s disease, APP: amyloid precursor protein.

**Table 3:** Characteristic and the transport mechanism of the main curcumin-conjugated nanocarriers for BBB.

Nano-Carrier Type	Shape/Size	Most Investigated Components	Mechanism in BBB	References
Solid Lipid Nanoparticles	Spherical (50–300)	Triglycerides, monoglycerides, complex glyceride mixtures, hard fats, cetyl alcohol, stearic acid, emulsifying wax and cholesterol butyrate. Surfactants are used to stabilise the lipid core (about 1–5% *w*/*v*) or co-surfactant (such as poloxamer 188 and/or Tween^®^ 80)	Uptake by the paracellular pathway through brain microvasculature, endocytosis and passive diffusion, tight junctions opening. Active targeting with apolipoprotein E	[156,157]
Liquid Crystalline Nanocarriers	Inverted hexagonal (hexosomes), bicontinuous cubic (cubosome), or sponge phases (20–200 nm)	Unsaturated monoglycerides, phospholipids, glycolipids and surfactants	Adsorption-mediated transcytosis, passive targeting or receptor-mediated endocytosis	[158,159]
Liposome	Globular/lamellar (20–200 nm)	Lipids: PEGylated 1,2-distearoyl-sn-glycero-3-phospho-ethanolamine-PEG 2000, phosphatidylcholine, ethyl-phosphatidyl-choline, 2-dipalmitoyl-sn-glycero-3-phospho-choline, lecithin, sphingomyelin, cholesterol,	Passive targeting, receptor-mediated endocytosis or Adsorption-mediated transcytosis Active targeting with receptors glutathione, glucose, transferrin, lactoferrin, apolipoprotein E, and phosphatidic acid	[160,161]
Micelles	Spherical (20–100 nm)	PLGA-PEG-PLGA triblock and PLGA-PEG diblock copolymers	Uptake by endocytosis and/or transcytosis. Targeting ligands with surface conjugation improve the transcytosis	[162,163]
Polymer Nanoparticles	Globular (10–200 nm)	PLGA, PBCA, PLA, chitosan and alginate.	Uptake by transcytosis and/or endocytosis through the endothelial cells and tight junctions opening	[132,156]
Cyclodextrins	Cyclic (150–500 nm)	Mainly the β-cyclodextrin derivatives	The direct action of cyclodextrin by extracting lipids like phospholipids and cholesterol, and proteins Modify the properties of the lipid bilayers and molecular composition	[164,165]

BBB: Blood-brain barrier, PLGA: Poly (lactic-co-glycolic acid), PEG: Polyethyleneglycol, PBCA: Poly(butyl)cyanoacrylate, PLA: poly (lactic acid).

## Abbreviations

AD	Alzheimer’s disease
APP	Amyloid precursor proteins
Aβ	Amyloid-beta
BBB	Blood-brain Barrier
CNS	Central nervous system
CUR/HP-β-CD inclusion complexes	Hydroxypropyl-β-cyclodextrin-encapsulated curcumin complexes
CUR-CS-PLGA-NPs	Curcumin encapsulated chitosan-coated PLGA nanoparticles
FCur NPs	Curcumin encapsulated Pluronic F127 nanoparticles
GBX	Gut-brain axis
GM	Gut microbiota
MAPK	Mitogen-activated protein kinase
NF-κB	Nuclear factor kappa-light-chain-enhancer of activated B cells
NFTs	neurofibrillary tangles
Nrf2	Nuclear factor erythroid 2-related factor 2
PBCA	Poly(butyl)cyanoacrylate
PEG	Polyethyleneglycol
PLA	Poly (lactic acid)
PLGA	Poly(lactic-co-glycolic acid)
ROS	Reactive oxygen species
WHO	World health organization
